# From neurons to nests: nest-building behaviour as a model in behavioural and comparative neuroscience

**DOI:** 10.1007/s10336-015-1214-5

**Published:** 2015-04-12

**Authors:** Zachary J. Hall, Simone L. Meddle, Susan D. Healy

**Affiliations:** 1 School of Biology, University of St Andrews, Harold Mitchell Building, St Andrews, KY16 9TH Scotland, UK; 2 The Roslin Institute, The Royal (Dick) School of Veterinary Studies, The University of Edinburgh, Easter Bush, Edinburgh, EH25 9RG Scotland, UK; 3 Department of Cell and Systems Biology, University of Toronto, Room RW618, 25 Harbord Street, Toronto, ON M5S 3G5 Canada

**Keywords:** Nest building, Neurobiology, Behavioural neuroscience, Motor sequencing, Comparative neuroscience

## Abstract

Despite centuries of observing the nest building of most extant bird species, we know surprisingly little about how birds build nests and, specifically, how the avian brain controls nest building. Here, we argue that nest building in birds may be a useful model behaviour in which to study how the brain controls behaviour. Specifically, we argue that nest building as a behavioural model provides a unique opportunity to study not only the mechanisms through which the brain controls behaviour within individuals of a single species but also how evolution may have shaped the brain to produce interspecific variation in nest-building behaviour. In this review, we outline the questions in both behavioural and comparative neuroscience that nest building could be used to address, summarize recent findings regarding the neurobiology of nest building in lab-reared zebra finches and across species building different nest structures, and suggest some future directions for the neurobiology of nest building.

## Introduction

Of all the constructions made by animals, perhaps none are as widely recognizable as the nests built by birds. From the gigantic mound nest of the Mallefowl *Leipoa ocellata*, in which eggs are incubated by the heat released from decaying wet vegetation buried within the nest (Frith 1959), to the cup-shaped nest of the Little Spiderhunter *Arachnothera longirostra* that is suspended from the underside of a banana leaf by strands of knotted vegetable fibres and spider silk forced upwards through the leaf to act as makeshift pop rivets (Hansell 2005), the nest building of birds has long fascinated us. This is evident in the collection of descriptions of nest structure for the majority of extant bird species gathered in the Handbooks of the World book series (e.g., del Hoyo et al. 1992). Given the diversity in nest building, it is perhaps surprising, then, that we know so little about how birds build nests.

To date, the investigative focus on nest-building behaviour has been directed at determining what role previous experience plays in nest building. Historically, nest building was assumed to be independent of experience with nest material and nests. For example, in "Descent of Man," Charles Darwin argued that inexperienced birds will construct nests comparable to those of experienced builders on their first attempt. In this account, he contrasted avian nest building with human motor skills, which typically improve with practice (Darwin 1871; but see Wallace 1867). Over the next eight decades, this view received relatively little support from experimental studies in which birds hand-reared in the absence of nest material and later exposed to nest material as adults, constructed nests resembling those built by experienced builders. For example, although female canaries *Serinus canaria* deprived of nest material in early life will still construct species-typical cup-shaped nests upon their first experience with nest material as adults (Hinde and Matthews 1958), similar manipulations in American robins *Turdus migratorius* and rose-breasted grosbeaks *Pheuticus ludovicianus* result in a failure to construct species-typical nests upon reaching adulthood (Scott 1902, 1904). Nicholas and Elsie Collias (1962, 1964, 1984) added a substantial body of work in which they documented the development of weaving abilities of African Village weaverbirds *Ploceus cucullatus*. They found that experience with nest material during development had a significant impact on the bird's subsequent nest material preferences and weaving abilities. One hundred and forty years on from Darwin and Wallace, there has been a surge in work on nest building in both free-living and captive birds, which is providing increasing experimental evidence for learning on selection of nest material (Muth and Healy 2011, 2012; Muth et al. 2013; Walsh et al. 2013; Bailey et al. 2014, 2015), nest structure (Walsh et al. 2010), nest location (Mennerat et al. 2009; Hoi et al. 2012), and building dexterity (Walsh et al. 2011).

Although this new body of research is beginning to make progress towards identifying the role that learning and memory may play in nest building, the entirety of this work addresses only one of the mechanisms that underpin nest-building behaviour, cognition. Here, we argue that studying the neurobiology of nest building offers a unique opportunity to investigate not only how the brain controls behaviour within single species and individual birds (behavioural neuroscience) but also how evolution has shaped the brain to produce interspecific behavioural variation (evolutionary neuroscience) using an ethologically relevant behaviour with significant fitness consequences in the wild. In this review, we outline the specific questions that the neurobiology of nest building could be used to address in each of these disciplines, summarize data from recent relevant experimental and comparative analyses, and propose directions for further research.

## Nest building and behavioural neuroscience

A central goal of behavioural neuroscience is the identification of the physiological mechanisms through which the brain controls different types of behaviour within an individual (Breedlove et al. 2010). We suggest that nest-building behaviour offers an opportunity to study the neurobiology of multiple types of behaviour, depending on the specific components of nest-building behaviour that are sampled. For example, by focusing on interactions between a pair of nest-building birds at the nest site, such as the time a pair of birds spend together in the nest (as in Hall et al. 2015) or duetting (Elie et al. 2010), one can investigate the neural substrates that may be involved in avian pair bonding and maintenance and the initiation of nest building. Or, by sampling the rate at which nest material is collected and brought to the nest and the rate at which the nest is built, we could use nest-building behaviour to study motivational processes involved in reproductively motivated behaviour. Additionally, as zebra finches building nests in captivity will change the way in which they handle nest material with their beaks (Muth and Healy 2011) and the types of nest material they select while building (Bailey et al. 2014) based on prior experience, nest-building behaviour could offer an opportunity to study the neurobiology of motor learning, in which birds change the physical actions they perform while building their nest, and physical cognition, in which birds learn the physical properties of materials with which they build. As at least some information about decisions made during nest building may be gleaned by examining the final nest structure (Collias and Collias 1964; Walsh et al. 2010, 2011), it may be possible to investigate the neurobiology of nest-building even in situations in which monitoring the entire construction process is not feasible. In the next section, we will focus on recent work in which we showed that nest-building behaviour also has potential to be an ethologically relevant model to study the neurobiology of motor sequencing and how this model could complement previous findings derived from food reward-based training studies.

Recently, we suggested that because nest building can be decomposed into sequences of discrete, organized motor actions, this behaviour offers an opportunity to study how the brain organizes discrete actions into motor sequences using a naturally occurring behaviour. For example, long-tailed tits *Aegithalos caudatus* construct domed nests that are composed of moss and up to 600 spider egg cocoons. Once most of the dome is built, the birds cover the outside of their nests with lichen flakes, which adhere in Velcro-like fashion to the spider silk incorporated into the nest walls. Finally, the birds create an entrance hole to the nest, complete the nest roof, and line the nest with an estimated 2600 feathers (Thorpe 1956; Hansell 2000). Tinbergen (1953) classified the nest-building process in long-tailed tits into 13–14 discrete, highly stereotyped actions that must be organized correctly to produce a viable nest. The correct sequence of nest-building actions is called the effective sequence, a term coined by Collias and Collias (1964) to describe the development of nest-building behaviour in African Village weaverbirds. Whereas the effective sequence of long-tailed tits and weaverbirds involves organizing multiple actions over long periods of time, nest building, in its simplest form, involves an effective sequence of collection of nest material and deposition of that material at the nest site.

Many current behavioural neuroscience models of sequence learning and motor sequencing rely on animals trained to respond to a series of stimuli in order to receive a food reward (serial reaction time tasks) or on animals trained to perform a series of actions using reward reinforcement (serial-order tasks; Schwarting 2009). In serial reaction time tasks, animals are trained to respond to individual stimuli presented in a sequence or randomized order. In a typical rodent test, the animal must poke its nose through one of five holes when the light above that hole is illuminated in order to receive a food reward. In the sequenced conditioning task, the five stimuli are presented in the same order on each trial, whereas in the control-conditioning task, the stimuli are presented in a randomized order (Schwarting 2009). The animal is assumed to have learnt the stimulus sequence when the reaction times to stimuli are lower in the sequenced condition task compared to the control condition, suggesting the animals in the sequenced condition can correctly predict the next stimulus in the sequence. Serial-order tasks typically involve operant conditioning procedures to train animals to press up to five buttons in a specific order. These paradigms have been used to compare motor sequence learning between humans, non-human primates, and birds (Scarf and Colombo 2008) and to identify neural substrates that may be involved in motor sequencing in the pigeon (Helduser and Güntürkün 2012; Helduser et al. 2013).

Although both serial reaction time and serial-order task paradigms provide accessible and robust training paradigms for investigating the neurobiology of motor sequencing, it is unclear how readily such data can be extrapolated to sequences of actions used in natural behaviours. For example, two features of both paradigms are that they rely on relatively short action sequences that occur over a few seconds and the repetition of the same action directed at different targets. In contrast, many of the action sequences that animals perform naturally occur over much longer timespans and can involve multiple different actions. Indeed the time to build a nest can vary from hours, days, to even weeks: for example, the Red-winged Blackbird *Agelaius phoeniceus* takes up to 3 days to construct a cup nest (Holcomb and Twiest 1968) while the male malleefowl constructs his nesting mound over the course of weeks and continues to maintain the nest daily for the majority of the year (Frith 1959). Comparing the neural substrates involved in nest building to those identified using the typical sequence training paradigms would, then, increase our understanding of how the brain organizes motor sequences across different timescales and action repertoires.

A second limitation of serial reaction time and serial-order tasks is that both rely on the provision of immediate and frequent food rewards in order to shape an animal’s behaviour, unlike many behaviours performed in the wild, including nest building. Importantly, the reward contingencies used during sequence learning in the laboratory may obscure the contributions of learning versus rewards to changes in task performance. For example, in serial reaction time tasks, animals performing the sequence-conditioning task typically become increasingly accurate over repeated trials (Schwarting 2009) and, thus, may receive a greater number of food rewards than do controls. This group difference in the quantity of rewards received over the entirety of task training can influence task motivation and, in turn, reaction times to respond to each stimulus, a common measure of performance in this paradigm (Schwarting 2009). Nest building could complement this training-based approach to studying motor sequencing as nest building does not rely on artificial food reward contingencies to change behaviour. Furthermore, in the absence of food reward contingencies, one could test whether the neural circuitry regulating the motivation and reward associated with ecologically-relevant behaviours such as courtship (O’Connell and Hoffman 2012) is also involved in the reinforcement of nest building.

## Looking for nest building in the zebra finch brain

The identification of the specific regions of the brain involved in a behaviour of interest is a common problem in behavioural neuroscience. One approach is to identify brain regions that are active while the subject performs the behaviour. Two of the most popular techniques for identifying patterns of brain activity in animals are blood-oxygen-level dependent functional magnetic resonance imaging (BOLD fMRI), in which increases in oxygenated blood flow, associated with increased neuronal activity, are measured (Ogawa et al. 1990) and electrophysiology, in which electrodes are implanted in a brain region of interest to measure neuronal activity in individual neurons or small groups of neurons while a behaviour is performed. Both fMRI and electrophysiological techniques have been adapted for use in bird species (Voss et al. 2007; Hahnloser et al. 2008), although fMRI recordings must be performed on an anesthetized or heavily restrained bird (Voss et al. 2007) leaving the animals unable to behave naturally. Electrophysiological techniques, on the other hand, require a specific candidate brain region to have been identified before electrodes can be implanted. An alternative methodology used to identify patterns of neural activity across whole brain divisions is to use immunohistochemistry or in situ hybridisation to label brain cells expressing immediate early genes. Immediate early genes are a group of genes expressed immediately following periods of changed activity in the cell (Clayton 2000; but see Kovács 2008 for other factors regulating immediate early gene expression). This technique, then, enables investigation of brain activation in awake, normally behaving animals.

One of the immediate early genes commonly used in behavioural neuroscience is *c*-*fos*, which is transcribed and translated to produce the protein product Fos (Morgan and Curran 1991). The appearance of Fos protein is time-dependent such that peak levels are expressed between 50 min to 2 h following elevated activity (Clayton 2000). This delay in Fos accumulation affords researchers an opportunity to allow an animal to perform the behaviour of interest freely without the use of anaesthetic or restraint and then to collect neural tissue to sample neuronal activity up to 2 h afterwards. Sampling the number of cells producing Fos protein in a given brain region provides an indirect measure of how active that brain region was during the time at which the behaviour was performed. Despite the reduced temporal resolution of Fos immunohistochemistry, attributed to the slow accumulation of Fos protein, data based on the localisation and quantification of Fos production in the vertebrate brain is a powerful technique for identifying candidate brain regions and, in combination with double labelling studies, the phenotype of the Fos-labelled cell. Once such brain regions have been identified, one can then employ techniques in which in vivo neuronal activity can be measured with high temporal resolution, such as electrophysiology. Specific immediate early gene studies have enabled the identification of brain regions exhibiting elevated neuronal activity in birds, and specific examples include during photostimulation (Meddle and Follett 1997), birdsong production (Kimpo and Doupe 1997), song perception (Bailey et al. 2002), sexual behaviour (Meddle et al. 1997, 1999), and social and agonistic interactions with conspecifics (e.g., Goodson 2005).

**Fig. 1:**
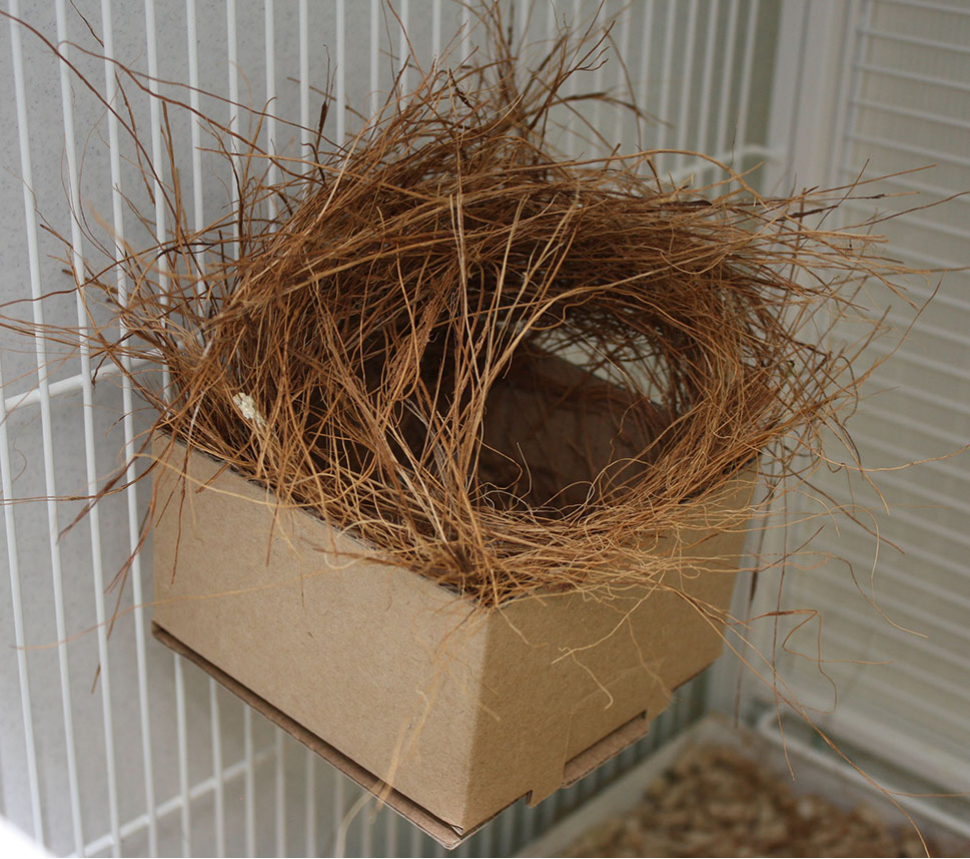
Photograph of a nest constructed by a pair of zebra finches in our laboratory. Photograph used with permission from Kate Morgan

In order to map which brain regions are active during nest building in birds, we employed Fos immunohistochemistry (Hall et al. 2014a, 2015). In our experiments, pairs of zebra finches *Taeniopygia guttata* were allowed to build a nest for 90 min, and the patterns of Fos production in the brain were compared to those of zebra finches that did not build a nest (Fig. 1). Zebra finches are a laboratory bird species commonly used to study the neurobiology of naturally occurring behaviour, particularly the production and perception of birdsong (Zeigler and Marler 2008). Importantly, male zebra finches readily build nests and breed under laboratory conditions when provided with a mate and nest material (e.g., Muth and Healy 2011). Typically, the male zebra finch collects and delivers nest material to the nest site while his female partner remains in or near the nest cup (Zann 1996; Hall et al. 2014a). Although both sexes then shape the material he brings to the nest cup, tucking new pieces into the growing structure, it is the male that primarily performs this manipulative task.

**Fig. 2:**
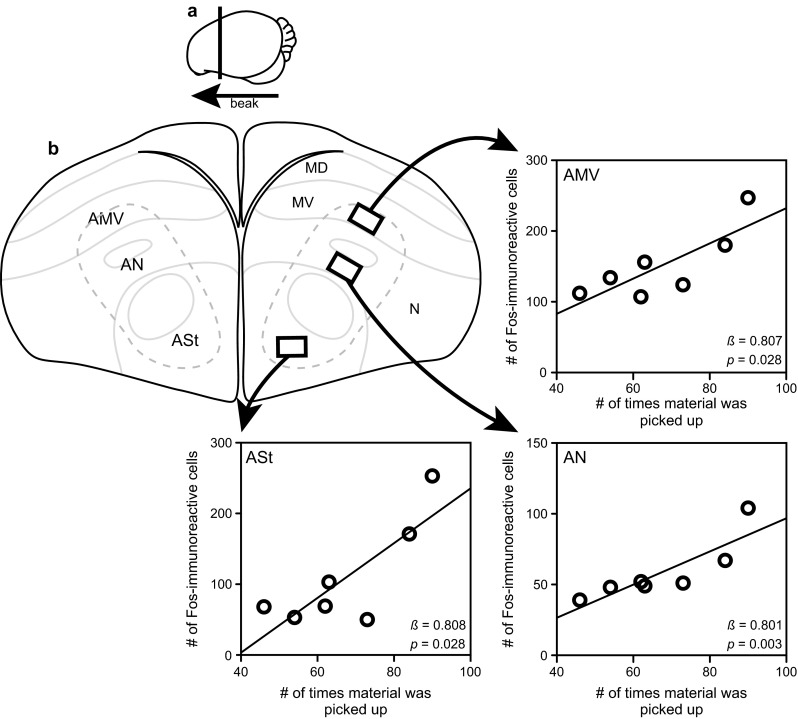
Increased Fos production in the anterior motor pathway in male nest-building zebra finches. A schematic of the sagittal (**a**) and coronal (**b**) locations of the anterior ventral mesopallium (AMV), anterior nidopallium (AN), and anterior striatum (ASt) of the anterior motor pathway sampled for Fos production in adult male nest-building zebra finches in Hall et al. (2014a). *Rectangles* in the right hemisphere depict sampling *squares* in AMV, AN, and ASt in which Fos production was quantified. *Arrows* from each sampling square point to the positive correlation between the number of times male finches picked up nest material 80–50 min prior to collection of neural tissue and the number of Fos-immunoreactive neurons sampled in each brain region. *MD* dorsal mesopallium, *MV* ventral mesopallium, *N* nidopallium

One of the neural circuits we identified as active during nest building was the anterior motor pathway (Hall et al. 2014a). In addition to the posterior motor pathway, the anterior motor pathway is one of two neural circuits in the avian forebrain thought to be involved in the production and organization of movement (Feenders et al. 2008). Based on the functions of brain regions located near each of these pathways, the anterior motor pathway (Fig. 2) is assumed to be involved in the organization and learning of actions while the posterior motor pathway (including the dorsal lateral nidopallium and lateral intermediate arcopallium) is thought to send signals through pre-motor brain regions to directly cause movement (Feenders et al. 2008). Consistent with the belief that nest building is organized into an effective sequence of nest material collection and deposition, neuronal activity in all three regions sampled in the anterior motor pathway in male nest-building finches increased the more these males picked up nest material to deliver to the nest cup (Fig. 2). As Fos production in this pathway increased, the more a male finch picked up nest material, which is the first behaviour in the effective sequence of nest building, it seems possible that the anterior motor pathway may be involved specifically with the initiation of motor sequences, including nest building. Similar neuronal activation in at least one region in the anterior motor pathway, the anterior nidopallium, has been demonstrated in pigeons performing a learned sequence of button pecks, suggesting this motor pathway is involved in all motor sequencing and not only nest-building behaviour (Helduser et al. 2012; Helduser and Güntürkün 2013). Neuronal activity in the posterior motor pathway was not, however, correlated with any nest-building behaviour and because both experimental and control birds could freely move and perform many non-nest-building activities. This finding is consistent with the suggestion that the posterior motor pathway is involved in the production of all movement, first proposed when neuronal activity in this pathway was found to positively correlate with locomotor behaviour performed by sensory-deprived birds (Feenders et al. 2008).

In addition to being a sequence of motor actions, nest-building behaviour in zebra finches is a reproductive behaviour often performed by a bonded pair of birds, which is regulated by a variety of social cues and motivational processes within a breeding context. To begin identifying the neural substrates that may be involved in the social modulation and motivation to build a nest, we also sampled Fos immunoreactivity in the social behaviour network and dopaminergic reward system, respectively. The social behaviour network is a group of interconnected brain regions involved in the production and regulation of social and reproductive behaviour in vertebrates (e.g., Goodson 2005). In birds, brain regions forming the social behaviour network are thought to be involved in producing reproductive behaviours, including courtship singing and displays (Heimovics and Riters 2006), copulation (Balthazart and Surlemont 1990; Meddle et al. 1997, 1999), aggressive interactions (Goodson and Adkins-Regan 1999), and incubation (Youngren et al. 1989). Furthermore, as neuronal activity increased in some regions of the social behaviour network in adult male starlings who possessed a nest box compared to males who did not (Heimovics and Riters 2005, 2006, 2007), this neural circuit may be involved in nest building as well. In zebra finches, we observed several different relationships between nest building and neuronal activity in the social behaviour network, including elevated Fos production in brain regions such as the mediodorsal division, the bed nucleus of the stria terminalis (BNST), and the preoptic area in nest-building birds compared to non-building controls (Hall et al. 2014a). We further demonstrated that increased Fos production in the mediodorsal BNST may be specifically attributed to a population of neurons that signal using the peptide hormone mesotocin (Hall et al. 2015). As Fos production in these brain regions did not change concomitantly with individual variation in nest-building behaviour, it seems that this increased Fos production may be related to the reproductive state associated with nest building, changes in other reproductive behaviours, or even to perception of the nest, rather than to a specific building behaviour itself.

In female zebra finches, neuronal activity in the medioventral division of the BNST increased the more time she spent in the nest (Hall et al. 2014a). This activity may reflect the female’s contribution to nest building, which can include receiving nest material and manipulating material to create the nest structure while at the nest site (Zann 1996), although more detailed analysis of behaviour within the nest is needed to determine whether this brain region is activated by in-nest building behaviour or is a response to the male’s building behaviour or even to physiology underlying her own impending egg laying. Activity in other brain regions within the social behaviour network decreased during nest building, suggesting that these regions play an inhibitory role (Hall et al. 2014a). Furthermore, when we sampled Fos production specifically in neuron populations in the social behaviour network that signal using the peptide hormones vasotocin and mesotocin, we found additional relationships between Fos production and nest building, including increases in neuronal activity in vasotocinergic neurons in the medioventral BNST the more a male finch spent time in the nest cup with his mate, suggesting this neural circuit may also be involved in the interaction between a pair of birds during nest building (Hall et al. 2015). At the very least, the pattern of changes in neuronal activity in the social behaviour network during nest building suggest that nest building should be included in the list of reproductive behaviours regulated by the social behaviour network.

In nest building male zebra finches, Fos production also increased in the dopaminergic reward neural circuit, specifically in the ventral tegmental area (Hall et al. 2014a) and in a population of neurons in the central gray that use the neurotransmitter dopamine (Hall et al. 2015). As noted above, the dopaminergic reward circuit is involved in motivational and reward processes that drive and reinforce behaviour in both reward-based learning paradigms in the laboratory and the production of social and reproductive behaviours in the wild (Riters 2011), often working in concert with the social behaviour network to achieve the latter (O’Connell and Hofmann 2012). In birds, regions within the dopaminergic reward circuit are involved in reward processes reinforcing courtship singing (Heimovics and Riters 2005), copulation (Charlier et al. 2005), affiliation behaviours (Goodson et al. 2009), and pair bonding (Banerjee et al. 2013). Components of the dopaminergic reward circuit also increased their activity in adult male starlings that possessed a nest box compared to males that did not (Heimovics and Riters 2005, 2007). It appears, then, that in addition to the social behaviour network, this neural pathway may be involved in the motivational processes to begin or continue building a nest.

Studies correlating behavioural performance to measures of neuronal activity in the brain, including Fos production in specific brain regions are, themselves, limited due to the low temporal resolution of immediate early gene techniques and because causation cannot be confirmed. They form the necessary basis, however, for future work, suggestions for which we describe in the next section.

## Future directions in behavioural neuroscience and nest building

The increases in Fos production we described above may reflect brain activity in regions that are involved in producing nest-building behaviour. Due to both the correlational nature and temporal resolution of Fos production in the brain, these increases in Fos production may also represent neuronal activity associated with sensory perception of the nest, non-specific motivational processes associated with breeding, or the performance of other, concurrent non-nest-building behaviours, such as hopping between the source of nest material and the nest site. Manipulation of neuronal activity with subsequent effects on behaviour and the use of techniques with high temporal resolution could help elucidate the specific roles that these brain regions and neuronal populations play in nest-building behaviour. For example, electrophysiological recordings in awake, behaving birds (e.g., Smulders and Jarvis 2013) could help test whether neuronal activity is associated with the production of a specific behaviour in the sequence of behaviours involved in building a nest. Pharmacological manipulations that temporarily reduce neuronal activity at the brain site of injection (Naie and Hahnloser 2011) complemented with subsequent observation of nest-building behaviour during suppressed activity could be used to demonstrate whether neuronal activity in a given brain region is necessary for the production of nest-building behaviour. Because many of the relationships between neuronal activity and nest building occur in neuron populations that use specific hormone and neurotransmitter signals including vasotocin, mesotocin, and dopamine (Hall et al. 2015), it would be possible to use gene silencing techniques or pharmacological agonism and antagonism (e.g., Tobin et al. 2010) to manipulate activity specifically within these chemical signalling pathways.

These suggestions for future studies are based directly on the data collected to date, and many other avenues remain completely unexplored in the study of the neurobiology of nest building. For example, the Fos work, as yet, has involved only zebra finches, in which males almost exclusively collect and deposit nest material. In some birds, it is the female or both female and male that collect and deposit nest material (e.g., in the common blackbird *Turdus merula*, it is the female that builds the nest; Ferguson-Lees et al. 2011). Whether sex differences in neuronal activity or neuroanatomy reflect sex roles during nest-building behaviour has yet to be addressed, and may be particularly interesting, as the vasotocin- and mesotocin-containing neuronal populations that we identified as active during nest-building behaviour (Hall et al. 2015) are sexually dimorphic across birds and other vertebrates (Goodson 2005). Similarly, zebra finches exhibit relatively simple nest-building behaviour consisting of collecting nest material, depositing material at the nest site, and tucking material to make a cup-shaped nest (Zann 1996). In contrast, species such as weaverbirds (Collias and Collias 1964) that can weave and thatch and learn to prefer long green strips of grass may be better suited to investigating the neurobiology of motor learning underlying fine motor control and material selection. In addition to the vasotocin, mesotocin, and dopaminergic systems we have already sampled, it seems likely that other hormone signalling pathways such as vasoactive intestinal polypeptide (VIP) play an important role in the modulation of nest-building behaviour. For example, neuronal activity in VIP-immunopositive neuron populations correlates with nest-building behaviour (Kingsbury et al. 2015), while prolactin under the control of VIP is important for brooding behaviour (Angelier and Chastel 2009) and plays an evolutionarily conserved role in parental behaviour across vertebrates. There is also evidence that steroids may also regulate the production of nest-building behaviour as female canaries treated with exogenous oestradiol expressed nest-building actions, even in the absence of nest material (Hinde and Steel 1976).

## Nest-building as a model in comparative neuroscience

Our understanding of how the brain controls behaviour is generally restricted to a few, intensively studied, typically laboratory-reared animal models. Our ability to generalize findings across a broad range of species may, then, be rather limited. Indeed, species-specificity of some brain-behaviour relationships may be the cause of the failure of, for example, neuropsychiatric therapeutic interventions that are first validated on laboratory animals and subsequently tested in humans (Hall et al. 2014b). By incorporating a wider range of species into neurobiological studies, we can reach a more holistic understanding of how the brain controls behaviour, including how data from a single species may reflect neurobiological processes in other species and taxa.

Currently, one major hindrance for comparative neuroscience is the lack of behavioural and neural data for large samples of species. Nest building may provide a useful source of such information, as descriptions of species-typical nest structure have been collected for the majority of extant bird species. Furthermore, it is possible that species-typical nest structure may reflect the manipulative nest-building behaviours a species exhibits while constructing the nest; however, the specific behavioural information that can be gleaned from a completed nest structure requires more observational work documenting the effective sequence of nest building during construction itself, akin to the observations made by Tinbergen (1953) on the long-tailed tit described above.

**Fig. 3:**
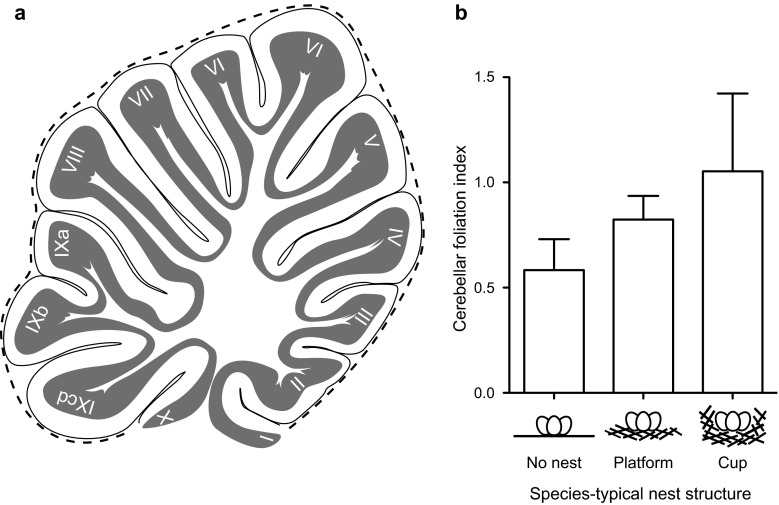
The relationship between cerebellar foliation and species-typical nest structure in birds. **a** A schematic of a sagittal section of the bird cerebellum. Cerebellar foliation was calculated by correcting the length of the surface length of the cerebellum (*grey*) for the surface length of a hypothetical unfolded cerebellum of the same size (*dashed line*; see Iwaniuk et al. 2006). **b** The average cerebellar foliation index (±SEM) for bird species that build either no nest, a platform nest, or a cup nest. Group differences were tested using phylogenetically-corrected regressions (Hall et al. 2013)

We recently tested whether species differences in nest structure reflect species differences in brain morphology within a structure we hypothesized may be involved in the manipulative skills birds use to build nests, the cerebellum (Hall et al. 2013). The cerebellum is a brain structure found in all vertebrates caudal to the forebrain, which was thought to serve only motor functions, including fine motor control. It is now known also to be involved in learning, memory, and, at least in humans, language processing (Barton 2012). In birds and mammals, cerebellar volume and the degree to which the surface of the cerebellum is folded (cerebellar foliation) are tremendously diverse across species (Larsell 1967). It has been suggested that expansion of the cerebellar surface and the associated increased cerebellar foliation increases the processing capacity of the cerebellum by increasing the number of neurons present in the surface layer, leading to improved motor control (Butler and Hodos 2005). Support for this hypothesis has come from evidence that cerebellar foliation is higher in birds that use tools than in birds that do not (Iwaniuk et al. 2009) and in primates that use extractive foraging techniques (Barton 2012), suggesting that increasing cerebellar foliation may specifically improve manipulative skill with the beak and hands in birds and primates, respectively. In a manner similar to tool manufacture and use, nest building appears to require variable degrees of manipulative skill to shape, stitch, and weave nest materials into different nest structures. Evidence to support this suggestion comes from the demonstration that cerebellar foliation increases with increasing complexity of the nest structure: birds that build nests of greater structural complexity (no nest→platform→cup) possess cerebella that are increasingly more foliated (Fig. 3; Hall et al. 2013). We suggest that these data support our proposal that nest building may be a useful system for investigating interspecific variation in neural correlates of behaviour. It should be noted that in our study, we addressed only a small section of the wide diversity in nest structure, ranging from the complete absence of nest material collection and deposition, such as in the arctic tern *Sterna paradisaea* that lays eggs in a simple ground scrape, to platform nests characterized by the collection of nest material into an unshaped pile, as is characteristic of a woodpigeon *Columba palumbus* nest, for example, to the collection of nest material and shaping of nest walls to produce a cup-shaped nest such as that of the American robin *Turdus migratorius*. But structural diversity in nests ranges well beyond this, to include domed nests, burrowing, the construction of entrance tunnels, weaving, and thatching using a wide array of materials. This provides ample and varied opportunity to study the neurobiology of motor learning and construction behaviour.

## Future directions in the comparative neuroscience of nest building

It is important to note that the correlation between cerebellar morphology and structural complexity of species-typical nests, as with all other correlations between behaviour and morphology, does not necessarily reflect a causal relationship between brain and behaviour. The next step, then, is to demonstrate that there is, indeed, a causal relationship between cerebellar function and nest building performance within individual birds. As the cerebellum is a large, multifunctional brain structure comprising individual surface out-folds, called folia—which are hypothesized to be functionally distinct and to contribute to motor control in different parts of the body (Iwaniuk et al. 2006)—nest building may not engage the entire cerebellum. This could be investigated by sampling neuronal activity in individual folia of the cerebellum, for example, by quantifying Fos protein production as described above, to determine which parts of the cerebellum are active during nest building.

Again, this future direction is a direct follow-up to the recent work, but there are many other promising avenues for comparative studies of the neurobiology of nest-building behaviour. For example, animals other than birds also build nests (invertebrates, lizards, fish, and mammals, e.g., Hansell 2005). As several of the neural substrates we have identified as involved in nest-building behaviour, including the striatum, cerebellum, vasotocinergic, mesotocinergic, and dopaminergic systems, are conserved across vertebrates (Reiner et al. 2004; O’Connell and Hofmann 2012), these substrates may be the place(s) to begin examining brain-behaviour relationships in nest building in other taxa. Furthermore, as nest building has been recognized as phenotypically similar to other construction behaviours such as tool manufacture and use (Hansell and Ruxton 2008), the neural processes that underlie nest-building behaviour may apply to other construction behaviours. Support for at least some shared neural processes comes from the demonstration that, within the same sample of birds, cerebellar foliation increases with both tool use and complexity of nest structure (Iwaniuk et al. 2006; Hall et al. 2013). Additionally, the striatum, which is part of the anterior motor pathway activated during nest building in birds (Hall et al. 2014a), also appears to be activated during tool use behaviour in primates, as measured using functional imaging (Obayashi et al. 2001). The apparent similarities between the neurobiology of nest building and other construction behaviours, however, require explicit testing, but it seems possible that at least some of the brain structures involved in construction behaviours have evolved to enable more general motor learning and control rather than specifically to enable/enhance nest-building or tool use behaviour.

## Nest building as an integrative model in neuroscience

Although work to elucidate the neural mechanisms involved in nest building is only just underway, we propose that nest building will prove to be a useful model in neurobiology. Rarely are we afforded the opportunity to complement invasive, mechanistic investigations, in which the physiological and molecular mechanisms underlying behaviour can be dissected in a single individual, with studies that span large samples of species, in which the functions of brain regions can be extrapolated beyond a single species. Nest building would, we believe, allow us to do this. Furthermore, the behavioural and comparative approaches to nest building do not exist as discrete research pathways but instead complement one another, providing direction for future work in both approaches. For example, examining patterns of Fos production in cerebellar folia during nest building in zebra finches, as suggested above, could help determine the specific role(s) the cerebellum may play in nest building and, thus, explain the relationship between cerebellar foliation and structural complexity of the nest (Hall et al. 2013). By continuing to refine our understanding of how the brain controls behaviour using mechanistic studies within single individuals and species and then testing how well these brain-behaviour relationships extrapolate to multiple species, we can achieve a robust understanding of the brain and identify the generality of neurobiological control of behaviour across species. This provides a context through which data from one animal model, such as nest building, may be transferred to other animals and even humans.
